# Cyp3A4 *1G polymorphism is associated with alcohol drinking: A 5-year retrospective single centered population-based study in China

**DOI:** 10.1371/journal.pone.0295184

**Published:** 2023-12-20

**Authors:** Xiaoqing Jia, Xiaoting Zhang, Tao Zhou, Dalong Sun, Rong Li, Na Yang, Zheng Luo

**Affiliations:** 1 Department of Gastroenterology, Qilu Hospital, Shandong University, Jinan, Shandong, China; 2 Department of Geriatric Medicine, Qilu Hospital, Shandong University, Jinan, Shandong, China; University of Ghana, GHANA

## Abstract

**Introduction:**

We investigated the epidemiology of Cytochrome P450 (CYP) 3A4 genotype and the relationship between CYP3A4 genotype and alcohol drinking habits.

**Materials and methods:**

A single-centered retrospective study was conducted on 630 patients who underwent CYP3A4*1G genetic testing. Their relevant information on epidemiology and etiology was collected. Laboratory testing, including CYP3A4*1G genotype, liver function tests, and serum lipid measurements were performed. Bi-variate logistic regressions were used to examine the relationship between variables. The relationship between alcohol drinking and CYP3A4*1G genotype was estimated. Demographic and clinical features were analyzed. Participants with drinking history were divided into non-heavy drinking and heavy drinking groups. Liver function and dyslipidemia of participants with drinking histories were compared between CYP3A4*1G mutation (GA+AA) and wild-type (GG) groups.

**Results:**

Participants with CYP3A4*1G mutation(GA+AA) had an increased adjusted odds ratio (AOR) of 2.56 (95% CI, 1.4–4.65; P = 0.00) for alcohol abuse when compared with participants without CYP3A4 mutation (GG). In the subgroup of participants with alcohol abuse, there are no significant differences in liver injury levels and serum lipid levels between CYP3A4*1G mutant and wild-type groups. Patients with CYP3A4*1G mutation had an increased AOR of cardiac-vascular diseases and malignant diseases compared with patients without CYP3A4*1G mutation. The epidemiology had no difference between GA and AA group.

**Conclusion:**

The study indicated that there was association between alcohol drinking and CYP3A4*1G genetic mutation. In the subgroup of participants with alcohol abuse, there are no significant differences in liver injury and dyslipidemia between CYP3A4*1G mutant and wild-type groups. CYP3A4*1G mutation was also related to cardiac-vascular diseases and malignant diseases.

## Introduction

Cytochrome P450 (CYP) is located in liver microsomes, which is indispensable in metabolizing a large group of clinical drugs [1]. Human CYP includes 18 families and 44 subfamily members [2]. CYP 3A4 metabolizes more than 50% of clinically used drugs [3–5]. Genetic factors are known to be responsible for the variability in the CYP3A4 activity [6, 7]. CYP3A4*1G (IVS10+12G >A, rs2242480) is a single nucleotide polymorphism site with a high mutation frequency in Chinese population, which includes wild-type homozygote (CYP3A4*1/*1, GG), mutant heterozygote (CYP3A4*1/*1G, GA), and mutant homozygote (CYP3A4*1G/*1G, AA) [8–10]. A synonymous G-A transition has been confirmed to be associated with CYP3A4 enzyme activity, which decrease metabolism of clinical drugs and requires distinct treatment among different patients [10, 11]. CYP3A4*1G genetic mutation has been found to decrease the activity of CYP3A4 which increase the fentanyl and sufentanil concentration in peripheral circulation and decrease the drug consumption for operative and postoperative pain control [12]. Researches have also shown that the genetic polymorphism of CYP3A4*1G has been associated with altered pharmacokinetics of several drugs, such as atorvastatin, tacrolimus, but may not be a significant contributor to clopidogrel metabolism [13–15].

Alcohol abuse is common in the general population. An estimated 2.4 billion people worldwide consumed alcohol, including 1.5 billion men and 900 million women in 2016 [16]. The harmful use of alcohol is responsible for 5.1% of the global burden of disease [17]. Chronic alcohol consumption is strongly related to several pathological consequences like liver injury, brain damage, type 2 diabetes mellitus, dyslipidemia and high blood pressure [18, 19]. Even low-level alcohol consumption could result in premature brain aging [20]. Common drinking patterns include light alcohol consumption, moderate alcohol consumption (MAC), binge drinking and chronic heavy drinking [19, 21, 22].

The alcohol-metabolizing in the human liver is a collection of multiple P450 species co-localized in the membrane of the endoplasmic reticulum (ER) [23]. The cytochrome P450 isozymes, mainly CYP2E1 contribute to alcohol oxidation in the liver and brain [24, 25]. Recent research indicates that alcohol exposure may induce CYP2E1-dependent activation of CYP3A4, CYP2E1-CYP3A4 interactions may play a role in the effects of alcohol exposure on drug metabolism in human liver microsomes [26]. But the clinical study on CYP3A4 activity and its association with alcohol drinking is limited. The association between polymorphisms of CYP3A4 genotypes and alcohol abuse is not clear.

Therefore, a retrospective population-based study was carried on to investigate the epidemiology of CYP3A4*1G genetic polymorphism and its association with alcohol drinking in China.

## Materials and methods

A retrospective observational study was conducted from January 2017 to December 2021at the Qilu Hospital of Shandong University in China. Sample size was calculated by the corresponding formula using PASS version 11.0 for windows. The ratio of alcohol abuse in control group was 18.95% in our research, and the expected odd ratio was 2 in a recent study (α = 0.05, β = 0.2) [27]. After calculation, the sample size of DILI group and the control group were at least 229. A total of 620 participants scheduled for surgery under general anesthesia in the Qilu hospital who underwent routine test of CYP3A4*1G genotype from January 2017 to December 2021 were enrolled in this study. More than two researchers participated in data collection and entry to reduce bias. Two researchers collected data simultaneously, then another researcher check the data to identify faulty or missing data and correct them. Incomplete data of participants were not included in the statistical analysis. Participants were recruited and medical records of participants were retrospectively collected from January 2017 to December 2021. Data assessment was carried on from December 2021 to December 2022. This study had access to information that could identify individual participants during data collection from medical records of participants in hospital. The Independent Institutional Review Board of Qilu Hospital of Shandong University authorized the recruitment of study participants and the study protocol number is KYLL-202008-097 (S1 and S2 Appendices). Data were collected after the ethic committee approval. All collected data is anonymous, and the requirement for informed consent was accordingly waived. The informed consent waiver statement was approved by the ethics committee of Qilu Hospital. All methods were conducted in accordance with the relevant guidelines and regulations. The raw data were supplied in S3 Appendix.

### Participants

Participants who underwent CYP3A4*1G genotype test at the Qilu Hospital of Shandong University from January 2017 to December 2021 were enrolled in the study. Participants less than 18 were not included in previous clinical study on CYP3A4 genotype [13, 15, 28, 29].

The exclusion criteria were as follows:

Under the age of 18;

Incomplete serologic results, demographic and clinical characteristics.

### CYP3A4*1G genotyping

The G-A synonymous transition of CYP3A4*1G genotypes were detected in the Department of Laboratory of Qilu Hospital. The blood samples were tested immediately after extraction or restored in 4 centigrade in refrigerator within 24 hours before genetic test. Genome DNA was isolated from 2 ml of peripheral venous blood with ethylenediaminetetraacetic acid as an anticoagulant and analyzed by using the TaqMan probe for genotyping. Primer sequence were as follows: F: 5’‑TGGTGAGGAGGCATTTTTGC‑3’ and R: 5’‑TGCAGGAGGAAATTGATGCA‑3’ according to previous researches [28]. PCR was carried out in a reaction mixture containing 0.4 μg of genomic DNA, 1.6 μmol/L of each primer, 0.25 μmol/L TaqMan probes and 1× PCR Mix (GeneCopoeia, Rockville, MD, USA). The PCR conditions included an initial denaturation at 95°C for 10 minutes, followed by 40 cycles of denaturation at 95°C for 10 seconds, annealing at 60°C for 30 seconds. The probe which contains a fluorescence reporter gene and a fluorescence quenching gene at both ends was digested and degraded by a 5’ nuclease of Taq enzyme and genotyping was quantified by the resulting fluorescence. All methods were carried out in accordance with the relevant guidelines and regulations of the Department of Laboratory of Qilu Hospital and previous researches [13, 15, 28].

Data were collected and analyzed, there were three genotypes, which include wild‑type homozygote (CYP3A4*1/*1, GG), mutant heterozygote (CYP3A4*1/*1G, GA), and mutant homozygote (CYP3A4*1G/*1G, AA). Participants were divided into two groups, which include CYP3A4*1G genetic wild-type group (GG) and CYP3A4*1G genetic mutant-type group (GA+AA).

### Classification of drinking patterns

Drinking patterns were divided into four classes [30]:

Light alcohol consumption: Regular alcohol drinking <10–20 g/day.

Moderate alcohol consumption: Regular alcohol drinking <30–42 g/day for men and <20–28 g/day for women

Binge drinking: Alcohol consumption >40 g for women and >50 g for men within about 2 h

Chronic heavy drinking: Chronic alcohol consumption (generally more than 5 years) more than 60 g on one occasion

In previous research, light alcohol consumption and moderate alcohol consumption belong to non-heavy drinking pattern [31]. Binge drinking is a pattern of heavy drinking in previous researches [32, 33]. In this study, participants with drinking history were then divided into non-heavy drinking group (light alcohol consumption and moderate alcohol consumption participants) and heavy drinking group (binge drinking and chronic heavy drinking participants). Participants with incomplete information of drinking quantity were excluded in the subgroup analysis.

### Diagnosis of dyslipidemia

Participants were considered to suffer from dyslipidemia if they had total cholesterol (Cho) >240 mg/dL or 6.00 mmol/L, high-density lipoprotein cholesterol (HDL-C) <40 mg/dL or 0.8 mmol/L, low-density lipoprotein cholesterol (LDL-C) ≥160 mg/dL or 3.37 mmol/L, or triglyceride (TG) ≥200 mg/dL or 1.7 mmol/L [34, 35].

### Data collection

The demographic and clinical characteristics evaluated in our study were collected from medical records in hospital, which include sex, age, smoking history, drinking alcohol history, allergy history, heart diseases, hypertension, type 2 diabetes, malignancy and history of family chronic disease. Biochemical parameters from routine laboratory tests of the patients were collected and analyzed, including alanine aminotransferase (ALT), aspartate aminotransferase (AST), Cho, HDL-C, LDL-C, TG and non-esterified fatty acids (NEFA).

### Statistical analysis

Percentages were used for qualitative parameters. Differences in proportions of drinking style between different genotype groups were evaluated using χ2-test. Mean ± standard deviation (mean ± SD) was used for normally distributed continuous variables including liver function levels and serum lipid levels in alcohol drinking participants. Spearman correlation analysis was performed on variables including sex, age, smoking, alcoholic abuse, hypertension, heart disease, diabetes, malignant disease, allergic history, family chronic disease and dyslipidemia to explore the relationship between variables and gene mutation. Bi-variate logistic regressions were used to investigate the relationship between genetic mutation and variables. Reference category of the exposure was designated of 1 to obtain OR value. We used multivariate analysis to select variables with P < 0.1 to adjust for potential confounding effects, entry method was used in selecting covariates in the multiple logistic regression model. Besides, sensitivity analysis were carried on which include multivariate logistic regression analysis of all participants’ information, the result were supported in supplementary data (S4 Apendix). Missing data were not included in statistical analysis. *P* value less than 0.05 was considered statistically significant. SPSS version 17.0 (SPSS, Chicago, IL) and GraphPad Prism 8.0 for Windows were used to perform statistical analysis.

## Results

### Characteristics of participants

**Fig 1:**
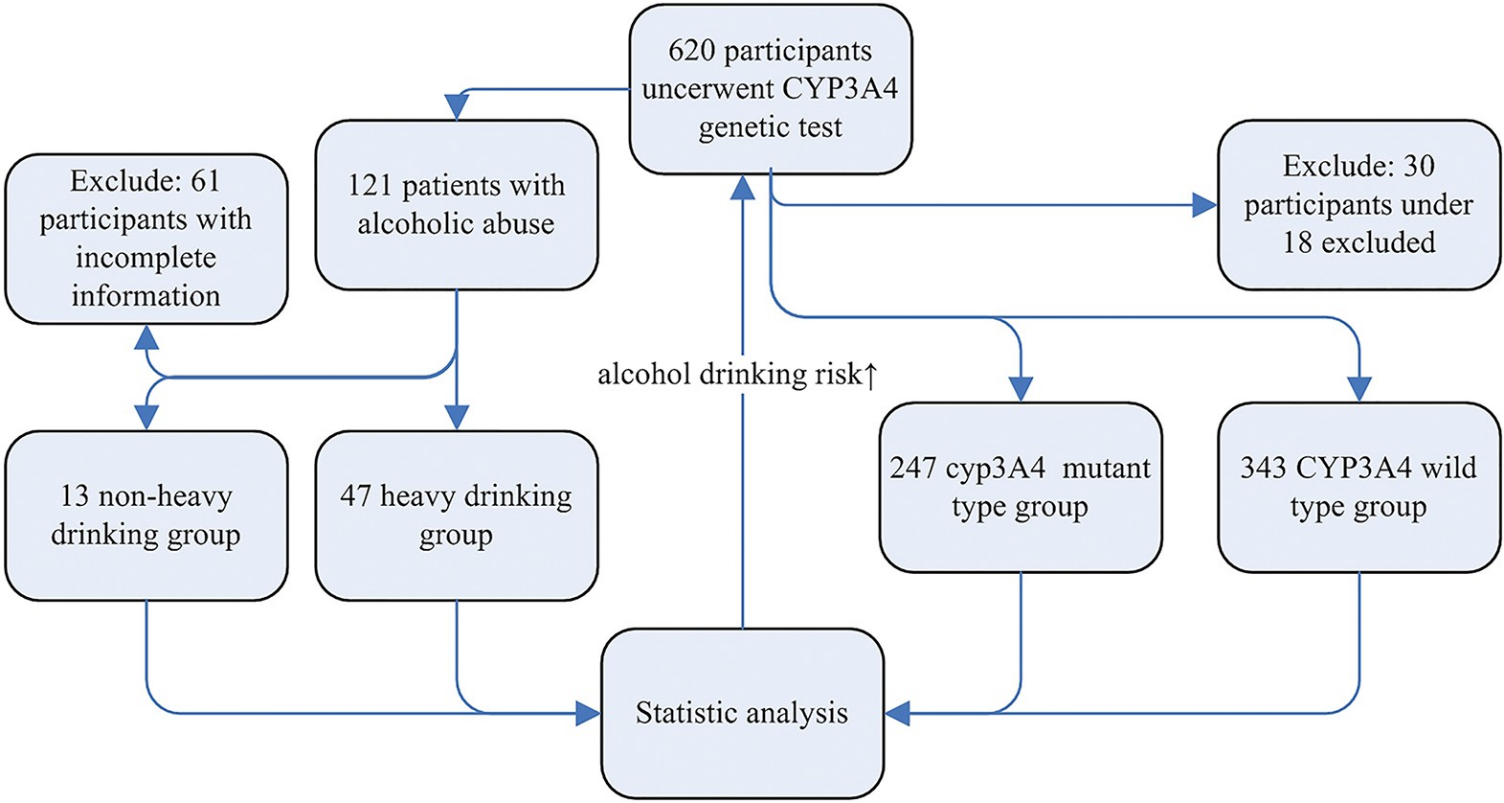
Study cohort. Participants under CYP3A4*1G genetic tests were included and separated into CYP3A4*1G mutant type and CYP3A4*1G wild type group. Clinical and epidemiology characters were analyzed and the result showed that CYP3A4*1G genetic mutation is associated with alcohol abuse. Participants with alcohol abuse were collected in subgroup analysis. The clinical and epidemiology characters were analyzed in alcohol abuse subgroup.

A total of 620 participants were enrolled in this study and 30 participants under 18 years old were excluded from the research. CYP3A4*1G mutant-type include mutant heterozygote (GA) and homozygote (AA), and there is no statistic difference between GA and AA in drug metabolism and epidemiology in previous study [13, 14, 28]. In the present study, all the individuals were divided into two groups according to the results of CYP3A4*1G genotype, including 343 with wild-type genotype (GG) and 247 with mutant-type genotype (GA+AA). The CYP3A4*1G mutant-type group includes 223 participants with mutant heterozygote (GA), and 24 participants with mutant homozygote (AA). In the CYP3A4*1G wild-type group, 55.98% were males and 44.02% were females; the median age of patients was 54.05 years. In the CYP3A4*1G mutant-type group, 56.28% were males and 43.72% were females; the median age of patients was 54.04 years. Demographic and clinical characteristics are shown in Table 1. Study cohort is shown in Fig 1.

**Table 1:** Patients’ characters in CYP3A4*1G mutant and wild-type groups.

variable		CYP3A4*1G wild type	CYP3A4*1G mutant type
sex			
	female (N%)	44.02	43.72
	male (N%)	55.98	56.28
age(mean±SD)		54.05±14.03	54.04±14.07
smoking			
	no (N%)	72.59	73.68
	yes (N%)	27.41	26.32
alcoholic abuse			
	no (N%)	81.05	74.49
	yes (N%)	18.95	25.51
hypertension			
	no (N%)	71.43	72.06
	yes (N%)	28.57	27.94
heart disease			
	no (N%)	80.47	75.71
	yes (N%)	19.53	24.29
diabetes			
	no (N%)	86.59	89.88
	yes (N%)	13.41	10.12
malignant disease			
	no (N%)	74.05	65.99
	yes (N%)	25.95	34.01
allergic history			
	no (N%)	89.8	89.88
	yes (N%)	10.2	10.12
familly chronic disease			
	no (N%)	91.84	89.07
	yes (N%)	8.16	10.93
dyslipidemia			
	no (N%)	63.27	64.78
	yes (N%)	36.73	35.22

### Clinical and demographic characteristics of CYP3A4*1G genotype

**Fig 2:**
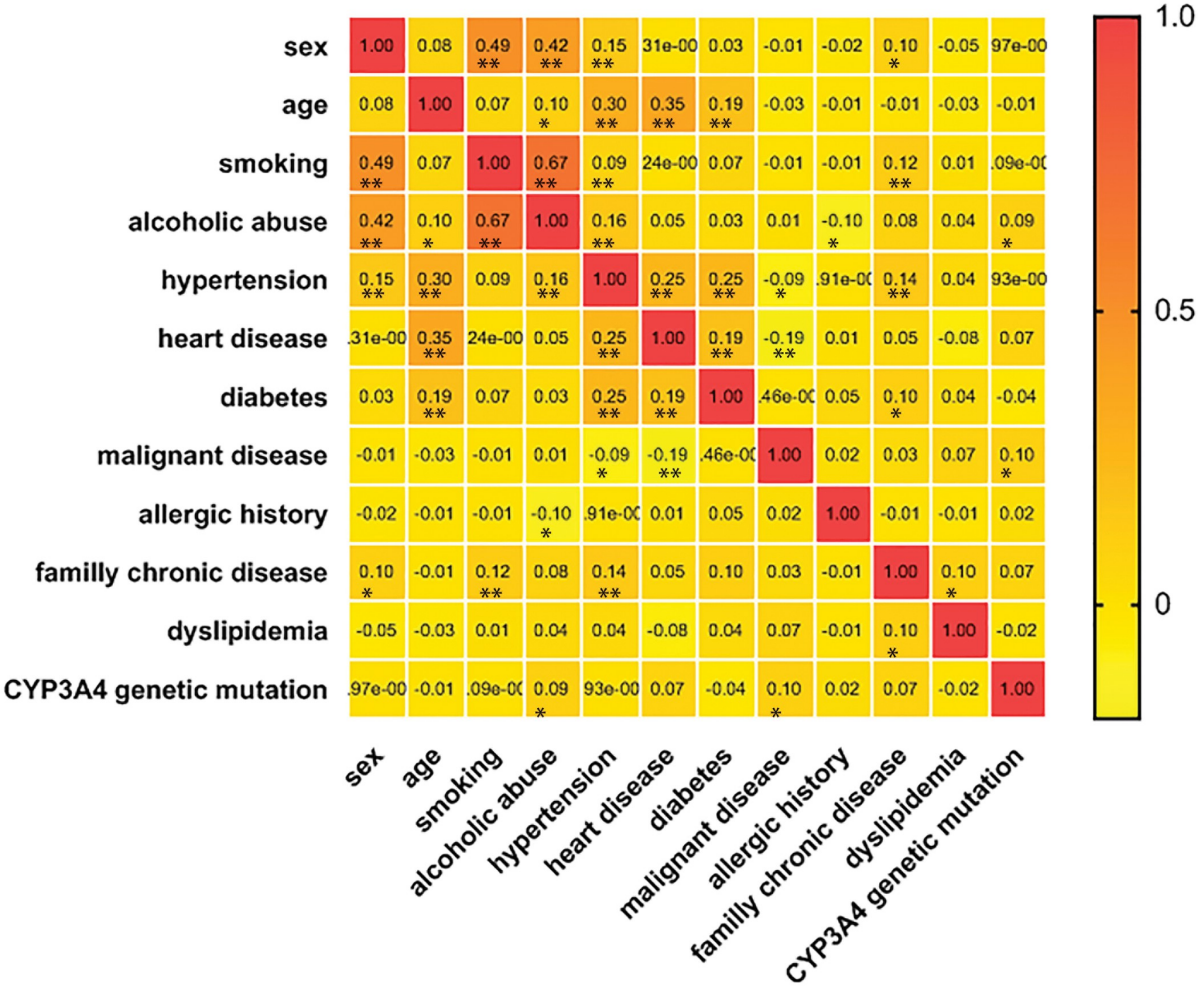
The spearman correlation coefficient between variables. The clinical and epidemiology characters were analyzed by Spearman correlation coefficient. * *P*≤0.05; ** *P*≤0.01.

CYP3A4*1G genetic mutation is associated with alcohol abuse and malignant diseases, with the correlation coefficient 0.09 and 0.1 (P<0.05) (Fig 2). Sex, age, smoking, allergic history, family chronic disease and CYP3A4*1G mutation were analyzed as exposure variables and alcoholic abuse, malignant disease, hypertension, heart disease, diabetes and dyslipidemia were analyzed as outcomes in logistic regression analysis. Study participants with mutant CYP3A4*1G had an increased odds (2.56 (1.4–4.65), *P* = 0.00) of alcohol drinking compared with those with wild type CYP3A4*1G, which indicated that participants with CYP3A4*1G genetic mutation had an increased incidence of alcohol drinking than patients without genetic mutation. Study participants of genetic mutant-type group with a history of cardiac vascular disease had an OR of 1.59 (95% CI, 1.03–2.46; P = 0.04) compared with those without genetic mutation, which showed that participants with CYP3A4*1G genetic mutation had an increased incidence of cardiac vascular diseases than patients without genetic mutation. CYP3A4*1G mutant-type group participants who had malignant diseases had an OR of 1.56 (95% CI, 1.08–2.24; P = 0.02) compared with those without genetic mutation, which indicated that participants with CYP3A4*1G genetic mutation had an increased risk of malignant diseases than patients without genetic mutation. There was no statistical significance association between hypertension, type 2 diabetes or dyslipidemia and the CYP3A4*1G genetic mutation. The epidemiology had no difference between CYP3A4*1G mutant heterozygote and homozygote. The above data are shown in Table 2. Multivariate regressions of all the participants were carried on and alcohol drinking still has increased OR in mutant-type group, the result was supplied in supplementary data (S4 Appendix).

**Table 2:** Univariate and bivariate analysis of patients’ characters.

variable	alcoholic abuse			malignant disease			hypertension			heart disease			diabetes			dyslipidemia		
	OR(95%CI)	AOR(95%CI)	*P*	OR(95%CI)	AOR(95%CI)	*P*	OR(95%CI)	AOR(95%CI)	*P*	OR(95%CI)	AOR(95%CI)	*P*	OR(95%CI)	AOR(95%CI)	*P*	OR(95%CI)	AOR(95%CI)	*P*
sex	34.49 (12.52–95.01)	8.45** (2.82–25.38)	0.00	0.95 (0.66–1.36)			1.99 (1.36–2.93)	1.87** (1.19–2.95)	0.01	1.02 (0.68–1.53)			1.21 (0.7–2.08)			0.82 (0.59–1.16)		
age	1.02 (1–1.03)	1.02 (0.99–1.04)	0.18	0.79 (0.54–1.15)			3.21 (2.2–4.69)	3.43** (2.32–5.08)	0.00	5.26 (3.37–8.2)	5.45** (3.48–8.52)	0.00	3.52 (2–6.18)	3.71** (2.09–6.56)	0.00	0.88 (0.62–1.24)		
smoking	40.7 (23.22–71.32)	24.99** (13.2–47.29)	0.00	0.97 (0.64–1.47)			1.54 (1.03–2.3)	1.05 (0.65–1.69)	0.85	1.02 (0.64–1.61)			1.57 (0.89–2.76)			1.06 (0.72–1.56)		
allergic history	0.29 (0.1–0.81)	0.16** (0.05–0.55)	0.00	1.14 (0.62–2.12)			0.99 (0.52–1.87)			1.06 (0.53–2.13)			1.63 (0.73–3.64)			0.91 (0.5–1.66)		
familly chronic disease	1.84 (0.96–3.51)	0.75 (0.3–1.89)	0.55	1.3 (0.69–2.44)			2.71 (1.49–4.94)	2.95** (1.56–5.59)	0.00	1.51 (0.77–2.96)			2.48 (1.17–5.28)	2.88** (1.31–6.32)	0.01	2.07 (1.15–3.75)	2.07** (1.15–3.75)	0.02
CYP3A4 mutation	1.59 (1.06–2.37)	2.56** (1.4–4.65)	0.00	1.56 (1.08–2.24)	1.56** (1.08–2.24)	0.02	1.01 (0.7–1.47)			1.43 (0.95–2.14)	1.59* (1.03–2.46)	0.04	0.75 (0.44–1.31)			0.94 (0.67–1.32)		
CYP3A4 mutant homozygote	1.23 (0.43–3.49)			0.7 (0.25–1.96)			1.5 (0.62–3.59)			1.15 (0.41–3.26)			0.41 (0.05–3.21)			0.64 (0.26–1.59)		

OR: odd ratio, AOR: adjusted odd ratio. The reference categories were designated as 1.00. The CYP3A4*1G mutant homozygote exposure was analyzed in AA group (1.00) compared with GA group (0).

* *P*≤0.05

** *P*≤0.01.

### Drinking patterns and CYP3A4*1G genetic polymorphism

Participants with drinking history were collected in subgroup analysis. The clinical and epidemiology characters were analyzed in alcohol abuse subgroup (Fig 1). Patients with were selected and divided into 4 patterns according to drinking patterns. Of 121 patients with drinking history, 60 patients with complete information were analyzed. Among patients with alcoholic abuse, 2 (3.33%) patients have light alcohol consumption, 11 (18.33%) patients have moderate alcohol consumption, 4 (6.67%) patients have binge drinking habits and 43 (71.67%) patients have chronic heavy drinking habits. Patients with alcoholic abuse were divided into non-heavy drinking (13, 21.67%) and heavy drinking (47, 78.33%) groups. There was no statistical significant association between CYP3A4*1G genetic mutation and drinking patterns (Table 3).

**Table 3:** Sub-group analysis at the level of the hepatic enzymes and serum lipid levels.

	N%		N%	*P* value	ALT (U/L)	AST (U/L)	Cho(mmol/L)	TG(mmol/L)	HDL-C(mmol/L)	LDL-C(mmol/L)	NEFA(umol/dl)
non-heavy drinking	21.67	CYP3A4*1G wild-type group	46.15	*P*>0.05	14±6.13	22.33±10.17	4.83±1.3	0.91±0.36	1.34±0.31	2.85±1.25	28.67±16.48
		CYP3A4*1G mutant-type group	53.85		52.29±74.08	53.43±77.99	5.57±3.47	1.41±0.66	1.12±0.51	2.61±0.7	51.86±23.54
heavy drinking	78.33	CYP3A4*1G wild-type group	48.94	*P*>0.05	33.3±41.92	29.96±30.53	4.59±0.89	1.3±0.61	1.19±0.2	2.85±0.72	37.57±40.58
		CYP3A4*1G mutant-type group	51.06		36.29±50.78	29.33±37.13	4.15±0.8	1.45±0.72	1.18±0.45	2.49±0.74	38.29±19.4

ALT: alanine aminotransferase, AST: aspartate aminotransferase, TG: triglyceride, Cho: cholesterol, LDL-C: low-density lipoprotein cholesterol, HDL-C: high-density lipoprotein cholesterol, NEFA: non-esterified fatty acids.

### Liver injury, dyslipidemia and CYP3A4*1G genetic polymorphism

Participants in the non-heavy drinking group had no statistically significant difference in ALT, AST, TG, Cho, HDL-C, LDL-C, NEFA levels between CYP3A4*1G wild-type and mutant-type. Patients in the heavy drinking group had no statistically significant difference in liver injury and dyslipidemia levels between CYP3A4*1G wild-type and mutant-type (Table 3).

## Discussion

CYP3A4 contributes to the metabolism of 45% of pharmaceutical drugs. Studies have shown that CYP3A4*1G genetic mutation decreases activity of CYP3A, resulting in a high plasma concentration of certain drugs like fentanyl and sufentanil [12, 28]. Previous studies have demonstrated that humans are primed to consume greater amounts of alcohol after a small dose, which is called rewarding effects [36]. Some drinkers who had greater sensitivity to these rewarding effects were prompt to have alcohol abuse, which is related to metabolism of alcohol in liver and blood alcohol concentration (BAC) [37]. The acetaldehyde is the oxidation product of ethanol which is generated by hepatic alcohol dehydrogenase (ADH) and the microsomal ethanol-oxidizing system (MEOS) [38]. The metabolism of acetaldehyde is mainly dependent on cytochrome P450 2E1 (CYP 2E1) [39]. Researches have shown that chronic alcohol exposure results in CYP3A4 activation through increased content of CYP2E1 which has high catalytic activity with ethanol, which indicated that CYP3A4 may play a role in ethanol metabolism [26, 40]. This study showed that participants with CYP3A4*1G mutation had an increased risk in the occurrence of alcohol drinking compared to participants with wild-type CYP3A4*1G genotype. The mechanism may be CYP3A4*1G mutation decrease the activity of CYP3A4 and CYP2E1 which increase BAC, therefore improve sensitivity to rewarding effects and neurotoxicity. However, there is no difference in liver injury between CYP3A4*1G wild-type and mutant-type in alcohol abuse sub-group.

This study showed that participants with CYP3A4*1G genetic mutation had a 1-fold to 2-fold increase in the incidence of malignant diseases than patients without genetic mutation. In recent research, CYP3A might play an important role in susceptibility to cancer, like leukemia, renal cancer and breast cancer [41–43]. Besides, alcohol consumption has a causative effect in a number of malignant tumors, such as cancers of the oral cavity, pharynx, larynx, esophagus, colorectum, liver, cervix and breast of female [44, 45]. The genetic mutation of CYP3A4*1G is supposed to be associated with malignant disease through the mechanism related to genetic mutation and alcohol abuse.

In this study, participants with CYP3A4*1G genetic mutation had a 1-fold to 2-fold increase in the incidence of cardiac diseases than participants without genetic mutation. Recent researches have shown that certain CYP450 enzymes contribute to cardiovascular diseases [46–48]. Alcohol consumption is related to increased risk of coronary artery disease, alcoholic cardiomyopathy, atrial and ventricular dysrhythmias, and hypertension, while high consumption or binge drinking significantly increases the risk [49]. The genetic mutation of CYP3A4*1G affects the activity of CYP3A4 and may be related to the susceptibility to cardiovascular disease. The pathology may be related to CYP3A4 activity and alcohol drinking.

Previous researches have demonstrated that CYP3A4 genetic mutation is associated with malignant diseases and cardiovascular diseases, which is in keep with the result of this study. The relationship between alcohol drinking and CYP3A4 genetic mutation has not been investigated in previous studies. This study has demonstrated that CYP3A4*1G genetic mutation has an increased risk of alcohol drinking.

### Study strengths and limitations

Compared with previous researches, firstly, this is the first work in China to study the correlation between CYP3A4 genetic mutation and alcohol drinking. Secondly, CYP3A4 genetic mutation, which may be associated with alcohol abuse, was related to heart diseases and malignant diseases, a regular check-up for specific population of alcohol abuse is essential. Thirdly, alcohol drinking which may be related to individual genotype should be understood and cared for, health education of quitting drinking as early as possible is essential.

This was a retrospective study that relied on the accuracy of medical records. Although our study has shown that CYP3A4 mutation is related to alcohol abuse, the mechanism is unclear, and the number of participants with drinking habits was limited, which may have potential bias. Clinical research needs to be improved by increasing sample size. Animal and molecular biology researches are needed to investigate the association between CYP3A4 genotype and alcohol drinking.

## Conclusion

Our research showed that CYP3A4*1G genetic polymorphism is related to alcohol abuse. In sub-group of participants with alcohol drinking, liver injury and dyslipidemia has no statistical significance between CYP3A4*1G mutation and wild-type group. The findings that have emerged can be utilized to further understand the epidemiological outcomes related to CYP3A4*1G mutation.

## Supporting information

S1 Appendix Ethic committee approval. (PDF)

S2 Appendix Ethic committe approval in English. (PDF)

S3 Appendix Raw data of the study. (XLSX)

S4 Appendix Sensitive analysis of multivariable logistic regression. (XLS)

## Abbreviations

CYP: Cytochrome P450
ALT: alanine aminotransferase
AST: aspartate aminotransferase
TG: triglyceride
Cho: cholesterol
HDL-C: high-density lipoprotein cholesterol
LDL-C: low-density lipoprotein cholesterol
NEFA: non-esterified fatty acids
