# Supplementary material for: Cyp3A4 *1G polymorphism is associated with alcohol drinking: A 5-year retrospective single centered population-based study in China
Source: PLoS One. 2023 Dec 20;18(12):e0295184. doi: 10.1371/journal.pone.0295184 (PMC10732449; doi:10.1371/journal.pone.0295184)
Supplement: S1 Appendix — (PDF) [file pone.0295184.s001.pdf]

山东大学齐鲁医院科研伦理委员会
伦理审查批件

伦理批件号	KYLL-202008-097		
项目名称	血脂异常与 CYP3A4 基因多态性及药物性肝病的相关性研究		
项目来源及编号	自筹		
研究科室	老年消化内科		
项目负责人	罗争		
审查类别	复审	审查方式	快速审查
审查日期	2020-12-25		
审查文件	复审申请<br>伦理申请表<br>豁免知情同意申请书<br>临床研究方案（V1.2 2020.8.14）<br>专家论证表		

审查意见：

根据我国《涉及人的生物医学研究伦理审查办法》、WMA《赫尔辛基宣言》和 CIOMS《人体生物医学研究国际道德指南》的伦理原则，经本伦理委员会审查，同意按所批准的文件开展本项研究。

1. 请遵循伦理委员会批准的方案开展临床研究，保护受试者的健康与权利。
2. 研究开始前，请申请人完成临床研究备案。
3. 研究过程中若变更主要研究者，对临床研究方案等的任何修改，请申请人提交修正案审查申请。
4. 发生严重不良事件，请申请人及时提交严重不良事件报告。
5. 请按照伦理委员会规定的年度/定期跟踪查频率，申请人在截止日期前 1 个月提交研究进展报告；当出现任何可能显著影响试验进行或增加受试者危险的情况时，请申请人及时向伦理委员会提交书面报告。
6. 申请人暂停或提前终止临床研究，请及时提交暂停/终止研究报告。
7. 完成临床研究，请申请人提交结题报告。

年度/定期跟踪审查频率	12 个月
有效期	20201225-20211225
主任委员签字	[signature]
伦理委员会	山东大学齐鲁医院科研伦理委员会（盖章）
日期	2020-12-25
