# Supplementary material for: Cyp3A4 *1G polymorphism is associated with alcohol drinking: A 5-year retrospective single centered population-based study in China
Source: PLoS One. 2023 Dec 20;18(12):e0295184. doi: 10.1371/journal.pone.0295184 (PMC10732449; doi:10.1371/journal.pone.0295184)
Supplement: S2 Appendix — (PDF) [file pone.0295184.s002.pdf]

Shandong University Qilu Hospital Research Ethics Committee

Ethics approval No. KYLL-202008-097

Study on the correlation between dyslipidemia and CYP3A4 gene polymorphism and drug-induced liver disease

Project source and serial number are self-financing

Research department geriatric gastroenterology

Project leader Luo Zheng

Review Type Review Mode Quick review

Review date 2020-12-25

Review document Review Application Ethics application form

Waiver of informed consent application

Clinical study protocol (V12 2020.8.14) Expert demonstration form

Review comments:

In accordance with the ethical principles of China's Approach to the Ethical Review of Biomedical Research Involving Humans, the WMA Declaration of Helsinki and CIOMS(International Ethical Guidelines for Biomedical Research on Humans), the Ethics Committee has reviewed and agreed to conduct this study in accordance with the approved documents.

1. Conduct clinical research in accordance with the protocol approved by the Ethics Committee to protect the health and rights of the subjects

2. Before the start of the study, the applicant should complete the clinical study record.

3. If the principal investigator is changed or any modification to the clinical study protocol is made during the study, the applicant shall submit an application for amendment review.

4. In the event of serious adverse events, the applicant is requested to submit a serious non-self-incident notification in a timely manner.

5. According to the annual/regular tracking frequency stipulated by the Ethics Committee, the applicant should submit the research progress report one month before the deadline; In the event of any situation that may significantly affect the conduct of the trial or increase the risk to

the subject, the applicant is requested to submit a written report to the Ethics Committee in a timely manner.

6. If the applicant suspends or prematurely terminates the clinical study, please submit the suspension/termination report in time.

7. Complete the clinical study and ask the applicant to submit the final report.

Annual/regular follow-up review frequency 12 months 12 months

Ethics Committee Shandong University Qilu Hospital Research Ethics Committee (seal)

2020-1225.
